# Sleep disturbances and sleep patterns in children with tic disorder: A case-control study

**DOI:** 10.3389/fped.2022.911343

**Published:** 2022-08-01

**Authors:** Yunhui Mi, Runzhi Zhao, Xiaoning Sun, Pingbo Yu, Wenqin Wang, Jijun Li, Zhenwen Liang, Hui Wang, Guanghai Wang, Kexing Sun

**Affiliations:** ^1^Department of Traditional Chinese Medicine, Shanghai Children's Medical Center, School of Medicine, Shanghai Jiao Tong University, Shanghai, China; ^2^Shanghai Hospital of Traditional Chinese Medicine, Shanghai University of Traditional Chinese Medicine, Shanghai, China; ^3^Department of Developmental and Behavioral Pediatrics, Shanghai Children's Medical Center, School of Medicine, Pediatric Translational Medicine Institute, Shanghai Jiao Tong University, Shanghai, China; ^4^Traditional Chinese Medicine, Children's Hospital of Fudan University, Shanghai, China; ^5^Shanghai University of Medicine and Health Sciences, College of Rehabilitation Sciences, Shanghai, China; ^6^Psychology Teaching and Research Section, Zhabei No.1 Central Primary School, Shanghai, China

**Keywords:** tic disorder, sleep duration, sleep disturbances, children, ADHD

## Abstract

**Study objectives:**

To characterize sleep disturbances and sleep patterns in children with Tic disorder (TD), and explore their association with TD severity and types.

**Methods:**

A case-control study was conducted in 271 children with TD recruited from a clinical setting and 271 non-TD children recruited from a primary school, matched by age (mean = 8.47 years, SD = 1.53 years) and gender (15.1% female). The Children's Sleep Habits Questionnaire (CSHQ) was used to assess sleep patterns and sleep disturbances. The TD types and severity were assessed with the Yale Global Tic Severity Scale (YGTSS).

**Results:**

The TD children scored higher on CSHQ total score than non-TD group (*t* = 29.50, *p* < 0.001) and demonstrated severer global sleep disturbance. Compared to non-TD children, TD children presented with increased risks for global sleep disturbance (aOR: 1.95; 95% CI = 1.20–3.06), and most specific sleep disturbances, including bedtime resistance (aOR: 3.15; 95% CI = 1.96–5.06), sleep onset delay (aOR: 3.43; 95% CI = 1.58–7.46), sleep anxiety (aOR: 2.83; 95%CI = 1.83–4.38), parasomnias (aOR: 3.68; 95% CI = 2.02–6.62), night waking (aOR: 9.29; 95% CI = 2.64–32.65), sleep disordered breathing (aOR: 1.72; 95% CI = 1.03-2.90) and daytime sleepiness (aOR: 1.72; 95% CI = 1.09–2.74). Children with mild and moderate tics, Provisional Tic Disorder (PTD), Chronic Tic Disorder (CTD) and Tourette Syndrome (TS) presented with more global and more specific sleep disturbances. In addition, combined ADHD, etc.

**Conclusion:**

Children with TD are major risks for increased sleep disturbances, especially for those with severe and chronic symptoms. Furthermore, comorbid ADHD increases risk in certain areas of sleep. These findings highlight the importance to consider sleep outcomes in the assessment and treatment for children with TD.

12pt

## Introduction

Sleep disturbances are common among general children and have been linked to a wide range of detrimental outcomes, including impairment in daytime functioning, emotional and behavioral difficulties, and comprised cognitive and academic performance (1–3). Emerging evidence has suggested that sleep disturbances occur more frequently in children with neurodevelopmental ailments such as Tic Disorder (TD) (4–6). For example, a recent systematic review revealed high prevalence of sleep disturbances on subjective measures such as parent-reported questionnaires in children with TD, ranging from 9.7 to 80.4% (5). In addition, previous studies using objective measures such as polysomnography mainly confirmed frequent sleep disturbances in children with TD (5, 7, 8). And some studies (9, 10) have found similarities in the mechanisms of TD and sleep disorders, highlighting that the two are intrinsically linked.

However, we found some gaps in further research on sleep disturbances in school-aged children with TD. The results of previous studies may not be completely consistent due to different data collection methods or diverse sample populations. For example, there is a study that sleep disturbance is related to the severity of TD (11), that is, the more severe TD will cause more severe sleep problems, and it is found that daytime sleepiness is common in moderate TD, while difficulty falling asleep, night waking and parasomnia are common in mild TD. In another study (12) with a larger age span, including children from preschool to adolescence, TD severity was a predictor of sleep deprivation interacting with gender and age. While the results were insightful, there was an obvious limitation of assessing sleep based on only a single outcome, the number of nights the child slept adequately in the past week. However, another one (4) suggest that there is no relationship between the two suggest that there is no relationship between the two. Such disparate findings and the multifaceted characteristics and associated factors of sleep disturbance in children with TD, such as the severity and type of tics, and the relationship to common comorbidities, remain largely unknown. Since there are demonstrated cross-cultural differences in sleep disturbances, such as the one between usually developing Chinese and American children with developmental disabilities, where have been reported more co-sleeping, shorter sleep and more sleep disturbances in general in the Chinese kids compared to the Americans, it may not be possible to generalize the existing relevant evidence (2, 13–15).

Therefore, based on the lack of research we found, in a well-defined clinical sample of children with TD as compared to age- and gender-matched non-TD children assessed with a validated and comprehensive sleep measure, the current case-control study aims to (1) characterize sleep disturbances and sleep patterns in Chinese children with TD; (2) explore whether the presence of TD, TD types, and severity were associated with increased risks for sleep disturbances and short sleep duration; (3) Sleep disturbances in children with TD, including abnormalities in different sleep dimensions, are associated with comorbidities ADHD.

## Methods

### Participants

The children in the TD group were outpatients who attended the Department of Traditional Chinese Medicine of Shanghai Children's Medical Center from February 2017 to December 2018. Experienced clinicians make TD diagnosis based on fifth edition of the Diagnostic and Statistical Manual of Mental Disorders (DSM-5) diagnostic criteria and comprehensive clinical evaluation. The data collection of all TD groups was completed at the first visit of the children, and the process was divided into two parts. First, the basic information required in this study and the sleep status of the children were obtained by means of questionnaires. Second, the Yale Global Tic Severity Scale (YGTSS) was assessed by trained clinical investigators in a quiet room at the first visit of the children to get the final score. By excluding children with severe medical conditions or who had been previously diagnosed with obsessive-compulsive disorder (OCD), depression, or other psychiatric disorders and children who had used psychotropic medication to control TD symptoms within 1 month prior to their first visit. We finally included all eligible Among the 271 children aged 6–11 years and there were more boys than girls (5.6:1). In order to exclude the influence of age and gender on the results of this study, the researchers stratified the children in the TD group and the non-TD group according to different ages (6, 7, 8, 9, 10, and 11 years old, respectively). According to the gender ratio of the included TD group children in different age groups, the same number was randomly selected 1:1 from the 731 non-TD children's data, and the cases of the same gender were included in the final study, that is, the non-TD group was also included in the final 271 cases.

This study was approved by the Ethics Committee of Shanghai Children's Medical Center, Shanghai Jiao Tong University School of Medicine (SCMCIRB-K2018027). All parents of children signed the informed consent form.

### Measures

#### Psychological tests

##### Children's sleep habits questionnaire

Sleep disturbances were assessed with the CSHQ (16). The CSHQ is a standardized and internationally recognized instrument consisting of 33 items. Parents rated the frequency of each item occurring in their children in the past week on a 3-point scale (usually, sometimes, and rarely). A total score of >41 indicates global sleep disturbances (17), covering eight domains with clinical cutoffs: bedtime resistance (10.84), sleep onset delay (2.31), sleep duration (5.27), sleep anxiety (7.79), night waking (5.29), parasomnias (10.61), sleep disordered breathing (4.50), and daytime sleepiness (15.24) (15, 18). The CSHQ reliability (Cronbach's alpha coefficient) of TD and non-TD children in this study were 0.60 and 0.62.

The CSHQ also investigated sleep patterns, including 3 items for bedtime, wake-up time, and daytime sleep duration on weekdays and weekends. Night sleep duration was calculated with sleep onset and wake up time on weekdays and weekends separately, and the total sleep duration included night sleep duration plus daytime sleep duration. Daily average total sleep duration was calculated as follows: [(weekday total sleep duration ×5) + (weekend total sleep duration ×2)]/7. According to the recommended sleep duration of 9–11 h per day for school-aged children (19). Therefore, in our study, it is believed that if the total sleep duration of school-aged children below the minimum standard (9 h) was considered as short sleep (20). Sleep arrangements, including bed-sharing, room-sharing, and sleep alone were also reported by parents.

##### Yale global tic severity scale

The YGTSS is a semi-structured clinical interview viewed as the gold standard for assessing the severity of tics in children and adults. The YGTSS evaluates the number, frequency, intensity, complexity, and interference of motor and phonic tics in the past week. Each domain is scored on a 6-point scale with a separate rating for “overall impairment” regarding the subject's daily life and activities. Five sum scores are available: the total motor tic score (range 0–25), the total phonic tic score (range 0–25), the total tic score (TTS; sum of the total motor tic score and the total phonic tic score), the overall impairment rating (one item; range 0–50), and the global severity score (GSS; sum of the TTS plus the overall impairment rating, range 0–100) (21). Two professionally trained graduate students conducted the YGTSS and produced a consensual score. Based on clinical manifestation and the YGTSS assessment, we dichotomized tic attack as mild or moderate/severe by defining a mild attack as a YGTSS score <20 and a moderate-to-severe tic attack as >20 (22).

According to the DSM-5 diagnostic criteria, TD is divided into three types: (1) Tourette's Syndrome (TS) characterized by the simultaneous appearance of sudden, short-term movement and vocalization symptoms before the age of 18. The symptoms last for more than 1 year; (2) Chronic Tic Disorder (CTD) is a single or multiple short-lived, rapid movements or vocal tics at a certain time during the onset of the disease before the age of 18, the two did not appear at the same time, the symptoms have lasted for more than 1 year and (3) Provisional Tic Disorder (PTD) is a single or multiple rapid, short-lived movement and/or vocal twitches discovered before the age of 18. The symptoms last <1 year. None of the above three types of tic symptoms are due to the direct physiological effects of drugs or other physical diseases (23).

#### Socio-demographic factors

Children's gender, age, family structure, and parents' educational attainment were reported. According to clinical experience, parents are more willing to provide this information does not involve the privacy of children and parents about wealth. Therefore, this study chose to collect this information.

The family structure included (1) extended family (live with both parents and grandparents), (2) core family (live with both parents), and (3) others (e.g., single parent or reorganized family). Caregiver's educational attainment was used as a proxy for socioeconomic status.

### Statistical analysis

Continuous data were presented with mean and standard deviation (SD). Categorical data were described with frequency and percentage (%). Comparisons between groups were performed using either chi-squared test (χ^2^-test) for categorical data or Student's *t* test (*t*-test) for continuous data. Then, conditional logistic regression model was used to calculate the unadjusted odds ratio (OR) and adjusted OR (aOR) for sleep disturbances and short sleep duration associated with the presence, types, and severity of TD, adjusting for socio- demographic factors, including age, gender, parents' educational attainment, and family structures. Since the two groups were strictly matched by age and gender, age and gender variables were not included in the analytical models. All analyses were performed using Statistical Package for Social Sciences (SPSS) software version 24.0 (IBM Corp., Armonk, NY, USA), with *p* < 0.05 being considered as statistically significant.

## Results

### Characteristics of participants

The descriptive characteristics of the participants were summarized in Table 1. For both groups, 84.9% were boys, and mean age was 8.47 ± 1.53 (range 6–11) years old. More mothers of TD children than non-TD children obtained education of senior high school or below (42.8 vs. 13.3%), but less obtained education of undergraduate (48.7 vs. 71.2%), and graduate or above (8.5 vs. 15.5%), with significant differences between the two groups (*p* < 0.001). The percent of TD children's fathers obtained education of senior high school or below than those non-TD children (44.6 vs. 12.5%), but less obtained education of undergraduate (48.3 vs. 77.5%), and graduate or above (7.0 vs. 10.0%), with significant differences between the two groups (*p* < 0.001). More TD children lived in a core family (53.5 vs. 43.2%), and less lived in an extended family (42.1 vs. 52.8%), with significant differences between the two groups (*p* < 0.05). The average YGTSS score of children with TD was 31.00 (±9.44), most of which were moderate (69.1%). For TD types, the most frequent was TS (45.4%), followed by CTD (31.7%) and PTD (22.9%).

**Table 1:** Descriptive characteristics of children with and without TD.

	**TD Children**	**Non-TD Children**		
	**(*****n*** = **271)**	**(*****n*** = **271)**	***t/***χ***2***	***p-*** **value**
**Age (years)**	8.47 ± 1.53	8.47 ± 1.53		
**Sex (*N*/%)**				
Boys	230 (84.9)	230 (84.9)		
Girls	41 (15.1)	41 (15.1)		
**Mothers' education level (*N*/%)**				
Senior high school or below	116 (42.8)	36 (13.3)		
Undergraduate	132 (48.7)	193 (71.2)	59.11	<0.001
Graduate or above	23 (8.5)	42 (15.5)		
**Father's education level (*N*/%)**				
Senior High school or below	121 (44.6)	34 (12.5)		
Undergraduate	131 (48.3)	210 (77.5)	68.53	<0.001
Graduate or above	19 (7.0)	27 (10.0)		
**Family structure (*N*/%)**				
Extend family	114 (42.1)	143 (52.8)		
Core family	145 (53.5)	117 (43.2)	6.31	0.04
Others	12 (4.4)	11 (4.1)		
YGTSS	31.00 ± 9.44	/		
**Tic severity**				
Mild	84 (31.0%)	/		
Moderate	187 (69.0%)	/		
**TD types**				
PTD	62 (22.9%)	/		
CTD	86 (31.7%)	/		
TS	123 (45.4%)	/		

YGTSS, yale global tic severity scale; TD, tic disorder; PTD, provisional tic disorder; CTD, chronic tic disorder; TS, tourette syndrome.

### Sleep arrangement and sleep patterns

Table 2 presents the comparison of sleeping patterns in TD and non-TD children. TD children were less likely to sleep alone or have room-sharing arrangement, but more likely to have bed- sharing arrangement than non-TD children (χ2 = 38.74; *p* < 0.001). Compared to non-TD children, sleep duration for TD children was longer on weekdays (9.52 ± 0.73 vs. 9.28 ± 0.66) and on average (9.65 ± 0.66 vs. 9.52 ± 0.56), but shorter on weekends (9.97 ± 0.88 vs. 10.11 ± 0.75). While TD children woke up earlier on weekends than non-TD children (7.63 ± 0.83 vs. 7.92 ± 0.75). There was no significant difference in the average wake-up time on weekdays, and there was no statistical difference in bedtime between the two groups of children, regardless of weekdays, weekend or average.

**Table 2:** Sleep arrangement and sleep patterns in children with and without TD.

	**TD children**	**Non-TD children**	**t/** *χ**2***	***p*** **-value**
**Sleep arrangement (** ***N*** **/%)**				
Sleep alone	85 (31.4)	121 (44.8)	38.74	<0.001
Room-sharing	50 (18.5)	83 (30.7)		
Bed-sharing	136 (50.2)	66 (24.4)		
**Total sleep duration (hours)**				
Weekdays, M ± SD	9.52 ± 0.73	9.28 ± 0.66	0.94	<0.001
Short sleep, *n* (%)	178 (45.2)	216 (54.8)	13.42	<0.001
Weekend, M ± SD	9.97 ± 0.88	10.11 ± 0.75	3.36	0.04
Short sleep, *n* (%)	111 (58.1)	80 (41.9)	10.44	<0.01
Average, M ± SD	9.65 ± 0.66	9.52 ± 0.56	4.68	0.01
Short sleep, *n* (%)	184 (45.9)	217 (54.1)	7.77	<0.01
**Weak-up time**				
Weekdays, M ± SD	6.83 ± 0.49	6.79 ± 0.36	3.67	0.26
Weekend, M ± SD	7.63 ± 0.83	7.92 ± 0.75	4.37	<0.001
Average, M ± SD	7.06 ± 0.49	7.11 ± 0.37	6.69	0.16
**Bed time**				
Weekdays, M ± SD	21.48 ± 0.64	21.56 ± 0.62	0.31	0.12
Weekend, M ± SD	21.90 ± 0.68	21.85 ± 0.65	0.46	0.45
Average, M ± SD	21.60 ± 0.58	21.65 ± 0.57	0.23	0.34

TD, tic disorder.

### Sleep disturbances

Table 3 presents the comparison of sleep disturbances in TD children and non-TD children. TD children scored significantly higher on the CSHQ total score (*p* < 0.001) and the subscales of bedtime resistance (*p* < 0.001), sleep onset delay (*p* < 0.001), sleep anxiety (*p* < 0.001), parasomnias (*p* < 0.001), night waking (*p* < 0.001), and sleep disorder breathing (*p* < 0.001), but not on the subscales of sleep duration and daytime sleepiness. The incidence of global sleep disturbances in TD children was higher than non-TD children (79.0 vs. 66.1%). Also, the incidence rates of bedtime resistance (52.8 vs. 19.2%), sleep onset delay (14.8 vs. 3.7%), sleep anxiety (45.4 vs. 21.4%), parasomnias (24.0 vs. 7.7%), night waking (10.3 vs. 1.1%) were higher in TD children. In the TD group, the highest incidence of subscale abnormalities was bedtime resistance (52.8%), followed by sleep anxiety (45.4%) and sleep duration (36.5%), and night waking was the lowest (10.3%). In the non-TD group, the highest incidence of subscale abnormalities was sleep duration (39.5%), followed by sleep anxiety (21.4%) and daytime sleepiness (19.6%), while night waking was the lowest (1.1%). Association between sleep disturbances and short sleep with TD symptoms.

**Table 3:** Sleep disturbances in children with and without TD.

	**TD children**	**Non-TD children**	***t/**χ**2***	***p-*** **value**
CSHQ total score, M ± SD	53.85 ± 7.20	51.27 ± 4.87	29.50	<0.001
*N* (%)	214 (79.0)	179 (66.1)	11.34	<0.05
Bedtime resistance, M ± SD	10.26 ± 2.95	8.34 ± 2.46	19.60	<0.001
Abnormal (*N*/%)	124 (52.8)	52 (19.2)	43.62	<0.001
Sleep onset delay, M ± SD	1.62 ± 0.74	1.27 ± 0.52	79.17	<0.001
Abnormal (*N*/%)	40 (14.8)	10 (3.7)	19.83	<0.001
Sleep anxiety, M ± SD	7.09 ± 2.23	5.77 ± 1.87	12.94	<0.001
Abnormal (*N*/%)	123 (45.4)	58 (21.4)	35.05	<0.001
Sleep duration, M ± SD	5.42 ± 1.11	5.49 ± 1.00	1.95	0.47
Abnormal (*N*/%)	99 (36.5)	107 (39.5)	0.50	0.54
Parasomnias, M ± SD	9.26 ± 2.21	8.32 ± 1.46	26.95	<0.001
Abnormal (*N*/%)	65 (24.0)	21 (7.7)	26.76	<0.001
Night waking, M ± SD	3.93 ± 1.23	3.36 ± 0.69	48.57	<0.001
Abnormal (*N*/%)	28 (10.3)	3 (1.1)	21.38	<0.001
Sleep disordered breathing, M ± SD	3.82 ± 1.24	3.56 ± 0.85	18.18	<0.001
Abnormal (*N*/%)	55 (20.3)	37 (13.7)	4.24	0.05
Daytime sleepiness, M ± SD	13.21 ± 3.20	13.27 ± 2.82	7.77	0.82
Abnormal (*N*/%)	70 (25.8)	53 (19.6)	3.04	0.10

TD, tic disorder; CSHQ, children's sleep habits questionnaire.

**Figure 1:**
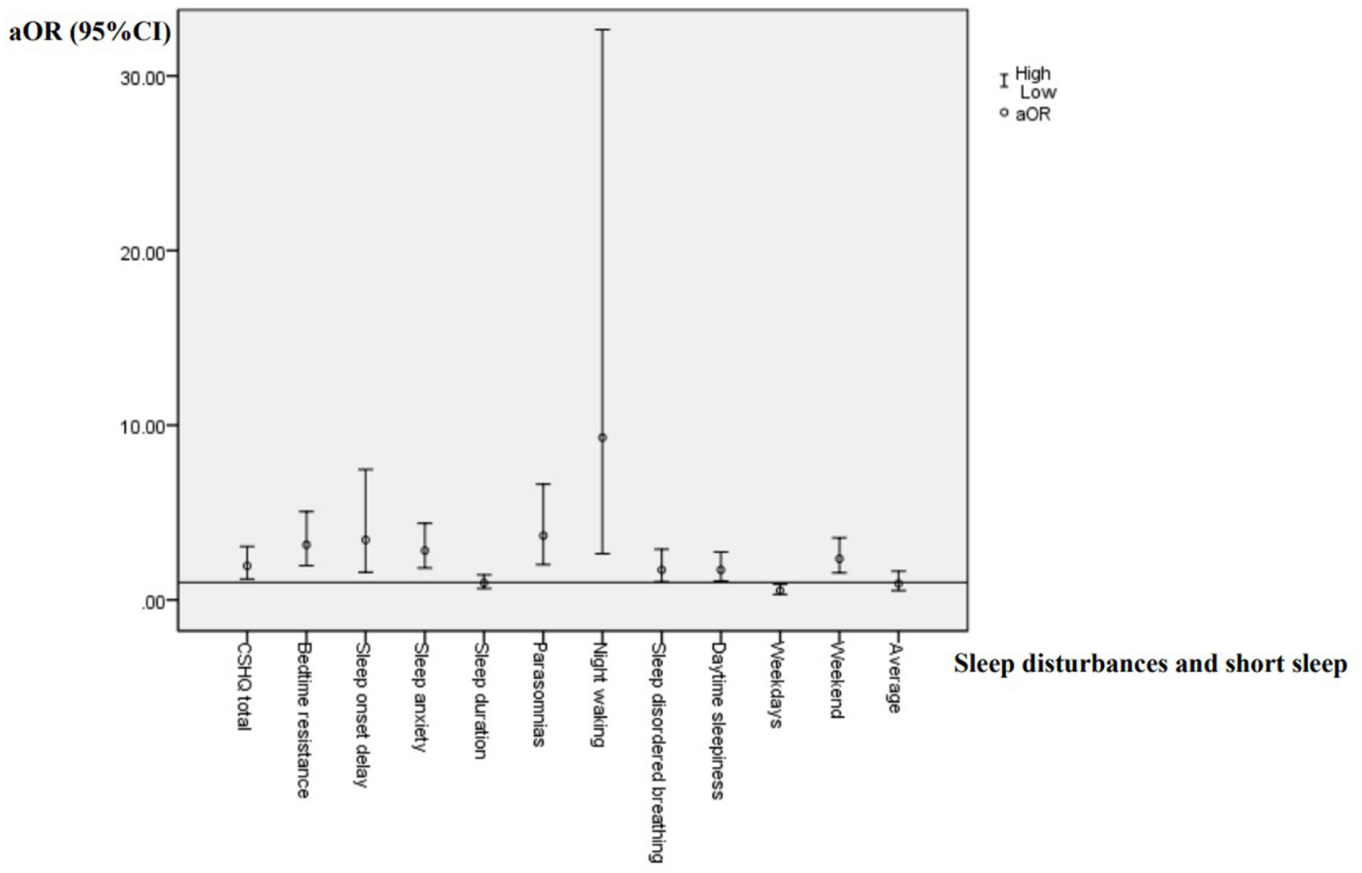
The association of TD with sleep disturbances and short sleep.

According to the variance inflammation factor and tolerance statistics, there is no complete linear relationship between the independent variables in any regression model. Presence of global and specific sleep disturbances, as well as short sleep duration were taken as dependent variables, respectively. Sleep arrangement, presence, types and severity of TD were entered as independent variables. Socio-demographic factors was entered as the covariates. Logistic regression analysis results are shown in Tables 4–**6** and Figure 1.

**Table 4:** The association of TD with sleep disturbances and short sleep.

	**Unadjusted OR, 95% CI**	**Adjusted OR, 95% CI**
CSHQ total	1.93 (1.31–2.84)^***^	1.95 (1.20– 3.06)^**^
Bedtime resistance	3.55 (2.42– 5.22)^**^	3.15 (1.96–5.06)^**^
Sleep onset delay	4.52 (2.21–9.24)^**^	3.43 (1.58–7.46)^**^
Sleep anxiety	3.05 (2.10–4.45)^**^	2.83 (1.83–4.38)^**^
Sleep duration	0.88 (0.62–1.25)	0.97 (0.65–1.44)
Parasomnias	3.76 (2.22–6.35)^**^	3.68 (2.02–6.62)^**^
Night waking	10.29 (3.09–34.29)^**^	9.29 (2.64–32.65)^**^
Sleep disordered breathing	1.61 (1.02–2.54)^*^	1.72 (1.03–2.90)^*^
Daytime sleepiness	1.43 (0.96–2.15)	1.72 (1.09–2.74)^*^
Short sleep		
Weekdays	0.47 (0.30–0.74)	0.54 (0.32–0.90)
Weekend	1.87 (0.68–5.13)	2.35 (1.55–3.56)^**^
Average	0.72 (0.44–1.16)	0.94 (0.54–1.65)

Non-TD children were used as the reference group. Adjusted for child gender, age, parents' education and family structure.

* p < 0.05,

** p < 0.01,

*** p < 0.001.

OR, odds ratio; CI, confidence interval; TD, tic disorder; CSHQ, children's sleep habits questionnaire.

According to Table 4 and Figure 1, children with TD had a greater risk of night waking (aOR: 9.29; 95% CI = 2.64–32.65), followed by parasomnias (aOR: 3.68; 95% CI = 2.02–6.62) and sleep onset delay (aOR: 3.43; 95%CI = 1.58–7.46). We further analyzed the effects of different severity and types of TD on sleep disturbances (Tables 5, 6). Compared with non-TD, night waking was associated with mild (aOR: 7.39; 95% CI = 1.75–31.22) and moderate TD (aOR: 10.02; 95% CI = 2.80–35.80), PTD (aOR: 8.33; 95% CI = 1.95–35.61), CTD (aOR: 5.80; 95% CI = 1.38–24.46), and TS (aOR: 12.17; 95% CI = 3.34–44.28). In addition, bedtime resistance, sleep onset delay, sleep anxiety, and parasomnias were all related to severity and types of TD (all *p* < 0.05). However, the shorter sleep on weekdays, weekends, or on average were not related to the severity and types of TD.

**Table 5:** Association of TD severity with sleep disturbances and short sleep.

	**Unadjusted OR (95% CI)**		**Adjusted OR (95% CI)**	
	**Mild**	**Moderate**	**Mild**	**Moderate**
CSHQ total	1.45 (0.84–2.50)	2.23 (1.43–3.48)^**^	1.46 (0.79–2.68)	2.24 (1.36–3.69)^**^
Bedtime resistance	4.02 (2.38–6.78)^**^	3.36 (2.21–5.11)^**^	3.89 (2.06–7.32)^**^	2.89 (1.75–4.78)^**^
Sleep onset delay	3.53 (1.41–8.80)^***^	4.99 (2.37–10.48)^***^	2.52 (0.94–6.77)	3.79 (1.71–8.41)^**^
Sleep anxiety	3.34 (1.99–5.60)^**^	2.93 (1.95–4.41)^**^	3.21 (1.79–5.74)^**^	2.69 (1.69–4.28)^**^
Sleep duration	0.81 (0.49–1.35)	0.92 (0.63–1.35)	0.91 (0.52–1.58)	1.00 (0.65–1.52)
Parasomnias	3.02 (1.51–6.05)^**^	4.11 (2.36–7.15)^**^	2.91 (1.36–6.21)^**^	4.00 (2.17–7.34)^**^
Night waking	8.12 (2.05–32.15)^**^	11.30 (3.32–38.48)^**^	7.39 (1.75–31.22)^**^	10.02 (2.80–35.80)^**^
Sleep disordered breathing	1.38 (0.71–2.65)	1.72 (1.05–2.82)^*^	1.48 (0.72–3.04)	1.82 (1.05–3.15)^*^
Daytime sleepiness	0.82 (0.43–1.57)	1.76 (1.14–2.71)^*^	1.01 (0.50–2.01)	2.07 (1.27–3.38)^**^
**Short total sleep duration**				
Weekdays	0.45 (0.22–0.90)	0.48 (0.29–0.79)	0.50 (0.23–1.07)	0.55 (0.32–0.96)
Weekend	1.64 (0.40–6.69)	1.98 (0.67–5.79)	1.22 (0.25–5.85)	1.62 (0.50–5.33)
Average	0.86 (0.43–1.72)	0.65 (0.37–1.14)	1.14 (0.54–2.45)	0.86 (0.46–1.59)

Non-TD children were used as the reference group. Adjusted for child gender, age, parents' education and family structure.

* p < 0.05,

** p < 0.01,

*** p < 0.001.

OR, odds ratio; CI, confidence interval; CSHQ, children's sleep habits questionnaire; TD, tic disorder.

**Table 6:** The association of TD types with sleep disturbances and short sleep duration (*N* = 271).

	**Unadjusted OR (95% CI)**			**Adjusted OR (95% CI)**		
	**PTD**	**CTD**	**TS**	**PTD**	**CTD**	**TS**
CSHQ total	1.36 (0.74–2.51)	2.43 (1.32–4.48)^**^	2.02 (1.22–3.34)^**^	1.21 (0.64–2.29)	2.26 (1.18–4.32)^*^	1.89 (1.10–3.25)^*^
Bedtime resistance	3.95 (2.21–7.07)^**^	4.21 (2.51–7.08)^**^	2.98 (1.87–4.77)^**^	3.55 (1.82–6.91)^**^	4.02 (2.17–7.46)^**^	2.75 (1.58–4.78)^**^
Sleep onset delay	6.26 (2.57–15.29)^**^	3.43 (1.38–8.56)^**^	4.47 (2.00–10.01)^**^	5.38 (2.13–13.58)^**^	2.92 (1.13–7.54)^*^	3.85 (1.66–8.98)^**^
Sleep anxiety	3.02 (1.70–5.39)^**^	3.85 (2.30–6.43)^**^	2.60 (1.64–4.13)^**^	2.67 (1.44–4.96)^**^	3.52 (2.01–6.15)^**^	2.38 (1.44–3.94)^***^
Sleep duration	1.04 (0.59–1.82)	1.05 (0.64–1.72)	0.71 (0.45–1.12)	1.05 (0.59–1.88)	1.08 (0.64–1.81)	0.73 (0.45–1.18)
Parasomnias	3.47 (1.65–7.30)^***^	4.34 (2.26–8.35)^**^	3.51 (1.90–6.48)^**^	3.07 (1.42–6.65)^**^	3.72 (1.88–7.38)^**^	3.11 (1.62–5.94)^***^
Night waking	9.57 (2.32–39.42)^**^	6.70 (1.64–27.39)^**^	13.36 (3.82–46.78)^**^	8.33 (1.95–35.61)^**^	5.80 (1.38–24.46)^*^	12.17 (3.34–44.28)^**^
Sleep disordered breathing	1.52 (0.74–3.12)	1.79 (0.97–3.32)	1.53 (0.87–2.70)	1.56 (0.74–3.28)	1.86 (0.97–3.56)	1.62 (0.885–2.95)
Daytime sleepiness	0.99 (0.49–1.98)	1.33 (0.75–2.37)	1.77 (1.09–2.88)^*^	1.12 (0.54–2.30)	1.48 (0.81–2.72)	2.04 (1.21–3.44)^**^
**Sleep duration**						
Weekdays	0.32 (0.13–0.78)^*^	0.44 (0.22–0.87)^*^	0.58 (0.33–1.01)	0.39 (0.16–0.98)	0.52 (0.25–1.07)	0.70 (0.39–1.26)
Weekend	1.47 (0.29–7.47)	1.60 (0.39–6.52)	2.27 (0.72–7.17)	1.40 (0.26–7.52)	1.50 (0.34–6.59)	2.09 (0.60–7.23)
Average	0.66 (0.28–1.54)	0.60 (0.28–1.29)	0.83 (0.45–1.52)	0.92 (0.38–2.21)	0.84 (0.38–1.86)	1.15 (0.60–2.19)

Non-TD children were used as the reference group. Adjusted for child gender, age, parents' education and family structure.

* p < 0.05;

** p < 0.01;

*** p < 0.001.

OR, odds ratio; CI, confidence interval; CSHQ, children's sleep habits questionnaire; TD, tic disorder; PTD, provisional tic disorder; CTD, chronic tic disorder; TS, tourette syndrome.

### Association between sleep disturbances and short sleep with comorbidity ADHD

According to Table 7, ADHD children and TD + ADHD children were found to have a higher risk of night waking than non-TD + ADHD children (aOR: 8.95; 95% CI = 2.45–32.66 vs. aOR: 9.91; 95% CI = 2.55–38.49, all *p* < 0.01). Among them, ADHD children had a higher risk of bedtime resistance (aOR: 2.93; 95% CI = 1.76–4.90, *p* < 0.001), followed by parasomnias (aOR: 2.66; 95% CI = 1.40–5.07, *p* < 0.01). For TD + ADHD children, the higher risk was parasomnias (aOR: 6.16; 95% CI = 3.12–12.18, *p* < 0.001), followed by sleep onset delay (aOR: 4.89; 95% CI = 2.07–11.56, *p* < 0.001).

**Table 7:** The association of ADHD with sleep disturbances and short sleep duration.

	**Unadjusted OR (95% CI)**		**Adjusted OR (95% CI)**	
	**TD (*****n*** = **172)**	**TD** + **ADHD (*****n*** = **99)**	**TD (*****n*** = **172)**	**TD** + **ADHD (*****n*** = **99)**
CSHQ total	1.64 (1.07–2.53)^*^	2.67 (1.48–4.82)^**^	1.60 (0.98–2.60)	2.96 (1.54–5.68)^**^
Bedtime resistance	3.41 (2.23–5.23)^***^	3.81 (2.32–6.26)^***^	2.93 (1.76–4.90)^***^	3.62 (1.97–6.64)^**^
Sleep onset delay	3.43 (1.57–7.53)^**^	6.61 (2.97–14.70)^***^	2.65 (1.14–6.17)^*^	4.89 (2.07–11.56)^***^
Sleep anxiety	2.91 (1.91–4.42)^***^	3.32 (2.03–5.42)^***^	2.63 (1.64–4.21)^***^	3.27 (1.87–5.70)^***^
Sleep duration	0.78 (0.52–1.16)	1.08 (0.68–1.73)	0.86 (0.55–1.33)	1.21 (0.72–2.02)
Parasomnias	2.72 (1.51–4.90)^**^	5.95 (3.23–10.96)^**^	2.66 (1.40–5.07)^**^	6.16 (3.12–12.18)^***^
Night waking	9.80 (2.83–33.97)^***^	11.17 (3.05–40.94)^***^	8.95 (2.45–32.66)^**^	9.91 (2.55–38.49)^**^
Sleep disordered breathing	1.62 (0.97–2.69)	1.60 (0.88–2.92)	1.70 (1.00–2.98)	1.78 (0.92–3.45)
Daytime sleepiness	1.41 (0.90–2.23)	1.47 (0.86–2.51)	1.71 (1.03–2.83)^*^	1.75 (0.97–3.18)
**Short total sleep duration**				
Weekdays	0.40 (0.26–0.61)^***^	0.72 (0.42–1.22)	0.48 (0.30–0.77)^**^	0.88 (0.49–1.58)
Weekend	1.42 (0.94–2.12)	2.16 (1.35–3.46)^*^	1.93 (1.22–3.03)^**^	3.32 (1.94–5.70)
Average	0.42 (0.27–0.65)^***^	0.82 (0.47–1.43)	0.52 (0.32–0.831)^**^	1.03 (0.56–1.90)

Non-TD and ADHD children were used as the reference group. Adjusted for child gender, age, parents' education and family structure.

* p < 0.05,

** p < 0.01,

*** p < 0.001.

OR, odds ratio; CI, confidence interval; CSHQ, children's sleep habits questionnaire; TD, tic disorder.

## Discussion

This study used an age- and gender-matched case-control design to characterize sleep disturbances and sleep patterns in school-aged children with TD, and explored the risks of sleep disturbances and short sleep linked to the presence, severity, and types of TD. Our results revealed that children with TD demonstrated more prevalent and severer sleep disturbances in global and most specific domains. Second, somewhat unexpectedly, we noted significant differences in sleep patterns between children with and without TD, such that TD children slept longer and had lower incidence of short sleep on weekdays, but slept shorter and had higher incidence of short sleep on the weekends. More importantly, this study found that increased risk of global sleep disturbances and several specific sleep disturbances (e.g., night waking, sleep onset delayed, and sleep anxiety) was associated with the presence of TD, and TD severity and types, but significant association was not found between short sleep and TD. And only-TD children and TD + ADHD children were found to have a higher risk of night waking than non-TD + ADHD children, with the difference being that only-TD children had a higher risk of bedtime resistance followed by parasomnias, and for TD + ADHD children, the higher risk was parasomnias, followed by sleep onset delay.

This study found that 79% of children in the TD group had sleep disturbances, a rate that is within the reported range of 7.24 to 80.0% in the literature (8). Our research results are consistent with previous studies on the effects of TD on children's sleep (5, 12, 24–32), indicating that TD children are globally and most certain areas show more severe and widespread sleep disturbances. A systematic analysis showed that more studies (66.7%) used subjective outcomes reported by parents (5). The results of one of the studies (25) were similar to ours, using the Sleep Disorders Questionnaire (SDQ) and finally found that children with TD were prone to difficulty falling asleep, anxiety, parasomnia, etc. This is similar to the dimension of abnormal sleep-in children with TD found in our study, except that this study also found more severe daytime sleepiness in TD, which was not found in our study. The research on polysomnography also mentioned the phenomenon that children with TD are not easy to fall asleep from an objective point of view (33, 34). Meanwhile, some studies (25, 35) found that children with TD had significant hyperactivity during the REM and NREM stages of sleep. This may be related to changes in neurotransmitters in children with TD, so they are more prone to nighttime awakenings, parasomnias, and anxiety. Therefore, although we did not use objective methods, such as polysomnography (PSG), our findings have credibility due to the similarity between our results and objectively assessed sleep outcomes. Some neurophysiological evidence (9, 33, 36–40) suggests a potential mechanism between TD and sleep disturbance. First, the ability of children with TD to control movement is related to the pathological involvement of cortical-striatal-thalamo-cortical (CSTC) disease in the brain (39), where the basal ganglia directly or indirectly affect the striatum to increase the excitability of the cerebral cortex (33, 38, 40). In addition, there is a class of views that TD patients have reduced 5-HT activity, and serotonin (5-HT) affects melatonin synthesis, which in turn leads to delayed sleep (9, 36, 37). Second, our findings suggest that TD children sleep longer on average per week and have lower rates of short sleep than non-TD children. This is not consistent with previous (12) findings that children in the healthy group received more adequate sleep per week than the TD group. At the same time, the analysis of this study shows that children with TD sleep longer during the weekdays and shorter during the weekend. Similar results have not been found in other studies, but some sleep studies (36, 41) of normal school-age children may explain this result. Typical school-age children have very short sleep duration on weekdays, so they tend to make up for sleep on weekends. The sleep of children with TD may be affected by certain external interventions. For example, their parents may encourage their children to go to bed early and get up late in their daily life to ensure longer sleep duration and these TD children need less sleep on weekends. Third, our results showed that the severity and types of TD were associated with risk of global sleep disturbances and several specific sleep dimensions. To be more specific, those with more severe TD symptoms were more likely to demonstrate sleep disturbances, particularly in terms of bedtime resistance, parasomnias, night waking, and sleep anxiety. This finding was consistent with previous studies showing more difficulties in falling asleep, night waking, and nocturnal activities in children with TD (12, 27, 42, 43). However, there are other contradictory evidences that show that sleep disturbances are not related to the severity of TD (5, 44, 45) and whether the severity of TD will affect sleep has not been conclusive. Some reasons, such as the heterogeneity of the age range, socioeconomic background, drug treatment, and other comorbidities of neurodevelopmental diseases and mood disturbances in different research populations, can cause this result to be ununified. Our study also showed that the presence of any types of TD was associated with increased sleep disturbances, especially for domains in bedtime resistance, sleep onset delay, sleep anxiety, parasomnias, and night waking, but there was no consistent pattern among different types of TD in relation to sleep disturbances. As this issue has only been limitedly explored, the current findings are critical to inform that the assessment and treatment for TD children should consider the accompanied sleep disturbances.

In fact, 99 patients (36.5%) in the TD group in our study had ADHD. ADHD is a common comorbidity of TD, and its high prevalence makes it difficult to ignore its impact on children's sleep. Based on previous studies (46), we reasonably speculate that the presence of ADHD may lead to more serious sleep disturbances. In a previous review (47), it was mentioned that about half of children with ADHD have sleep disturbances, which are manifested in increased latency to fall asleep, shorter sleep duration, more parasomnias, and increased overall sleep problems. We also further analyzed the sleep conditions of non-TD children, TD, TD + ADHD children, and found similar results to the above research, that is, compared with the effect of TD on sleep, if children with comorbid ADHD, it will increase its risk of sleep disturbances is reflected in the sleep duration, bedtime resistance, delayed falling asleep, sleep anxiety, parasomnias, night waking, etc. Another study used polysomnography to provide objective evidence support, that is, normal children, TD, ADHD, TD + ADHD children's sleep processes were recorded, and the results also found that children with only ADHD or TD with ADHD showed more awakenings and repeated sleep interruptions. The study (48) also believes that children's behavioral problems will affect sleep to a certain extent, and repeated awakenings during sleep will affect their daytime behavior. It may be a process of mutual influence, but it still lacks in-depth research.

## Strengths and limitations

This study is among the first to explore sleep disturbances and sleep patterns associated with TD in Chinese school-aged children. As we utilized a strictly age- and gender-matched case-control study design, the comparison between TD and non-TD children is believed to be more accurate and robust. Furthermore, the current investigation into the association between sleep and TD in school-aged children considered as many phonotypes of sleep and TD as possible.

This study also has several limitations. First, the data for TD children was from one site. Therefore, the generalizability of our findings to other populations may be limited. Thus, further multi-center studies, particularly those with larger and more representative samples, are warranted. Second, we only used parent-reported questionnaire to investigate children's sleep conditions, and this is subject to recalling bias. Nevertheless, the CSHQ is a validated and standardized sleep measure that has been widely used in typically developing children and children with neurodevelopment disorders, and demonstrates acceptable agreement with objective measure of actigraphy. Third, the current study is cross-sectional in nature, and thus cannot infer causal relationship between TD symptoms and sleep disturbances. Longitudinal and intervention studies may shed more light on this aspect. Further, due to the limitations of clinical data collection, some factors that may be related to sleep have not been collected completely, which will be the part that needs to be improved in the follow-up research. The last, our study only included TD children with comorbid ADHD, while children with comorbid OCD and depression were excluded from this study, so the inclusion of more comorbidities may be a research gap that needs to be addressed in the future.

## Conclusions

As the research progresses, more and more evidences can show that children with TD are more common sleep disturbances. While reporting the same results, our study proposes that children with TD have a higher risk of night waking, parasomnias, sleep onset delayed. Despite the above results, based on a deeper consideration of the relationship between the severity and types of TD and increased sleep disturbance, the results suggest a greater risk of night waking, parasomnias, sleep onset delayed, and sleep anxiety. However, there are few similar studies, not only lack of objective research results, but also lack of subjective research results, although subjective results are easier to obtain, Therefore, we can also pay attention to this issue from a more objective point of view, and continue to use subjective questionnaires to collect more relevant data and conduct more data analysis of the results. However, our findings still underscore that we need to attach importance to sleep problems when evaluating and treating children with TD, and considerate the impact of comorbidities on sleep among children with TD as appropriate as we can.

## Data availability statement

The raw data supporting the conclusions of this article will be made available by the authors, without undue reservation.

## Ethics statement

The studies involving human participants were reviewed and approved by Ethics Committee of Shanghai Children's Medical Center. Written informed consent to participate in this study was provided by the participants' legal guardian/next of kin.

## Author contributions

YM and RZ jointly contributed to the conception of this manuscript. RZ reviewed the relevant materials. YM analyzed and interpreted the data, drafted the manuscript, and agreed to take responsibility for all aspects of the work to ensure that issues related to the accuracy or completeness of any part of the work were properly investigated and resolve. XS, PY, WW, JL, ZL, HW, GW, and KS have made significant contributions to the concept or design of this study or this manuscript, made critical revisions to the manuscript, approved the final version for publication and assisted in the investigation, and resolution of issues related to accuracy. All authors contributed to the article and approved the submitted version.

## Funding

This study was supported by the fund of Shanghai Administration of Traditional Chinese Medicine (ZHYY ZXYJHZX-201918) [ZY(2021-2023)-0206-08], Shanghai Municipal Health Commission (2020LP022), Shanghai Children's Medical Center (LY-SCMC2020-03), National Natural Science Foundation of China (82071493, 81773443, and 82103866), and Shanghai Science and Technology Commission (18JC1420305, 19QA1405800, and 19411968800).

## Conflict of interest

The authors declare that the research was conducted in the absence of any commercial or financial relationships that could be construed as a potential conflict of interest.

## Publisher's note

All claims expressed in this article are solely those of the authors and do not necessarily represent those of their affiliated organizations, or those of the publisher, the editors and the reviewers. Any product that may be evaluated in this article, or claim that may be made by its manufacturer, is not guaranteed or endorsed by the publisher.
